# Extreme lateral interbody fusion (XLIF) in a consecutive series of 72 patients

**DOI:** 10.17305/bjbms.2020.5261

**Published:** 2021-10

**Authors:** Mirza Pojskić, Benjamin Saβ, Benjamin Völlger, Christopher Nimsky, Barbara Carl

**Affiliations:** 1 Department of Neurosurgery, University of Marburg, Marburg, Germany; 2 Marburg Center for Mind, Brain and Behavior (MCMBB) Marburg, Germany; 3 Department of Neurosurgery, Helios Dr. Horst Schmidt Kliniken, Wiesbaden, Germany

**Keywords:** Extreme lateral interbody fusion, retroperitoneal approach, spinal canal stenosis, spondylodiscitis, non-fusion, multilevel spinal surgery

## Abstract

Extreme lateral interbody fusion (XLIF) has become the standard of minimally invasive lumbar segmental scoliosis treatment. Our objective is to determine the safety and efficacy of XLIF in spinal canal stenosis (SCS) and spondylodiscitis (SD). Patients treated with XLIF in our department between 2012 and 2018 were retrospectively analyzed. Patient records with clinical and radiographical parameters were evaluated. The patient cohort consists of 40 male and 32 female patients with a median age of 66.6 years. Forty-five patients had an SCS and 27 patients SD. The mean follow-up was 23 months. One level XLIF was performed in 49 patients, 2 levels in 15, 3 levels in 7 patients and 4 levels in 1 patient. All but one patient received an additional dorsal stabilization. The pain was present in all patients with a mean visual analog scale (VAS) score of 8.8 versus postoperative VAS of 2.8 (p < 0.05). Preoperative neurological deficits were found in 44 patients. Only 6 patients had a neurological deterioration, 45 patients improved, and 21 patients remained unchanged. One patient experienced a perioperative complication. Non-fusion occurred in 8 cases. There were no outcome differences regarding pain and radiological outcome between patients with SCS and SD as well as between patients with one level vs. multilevel surgery. Baseline characteristics and the radiological outcome did not differ between the two groups. Patients with SD had a higher rate of worsening of neurological deficits following surgery, a higher rate of non-fusion, and a longer hospital stay. Patients with spinal canal stenosis SCS had a longer surgery time and more frequent adjacent segment disease.

## INTRODUCTION

Extreme lateral interbody fusion (XLIF) has become the standard of minimally invasive therapy of the degenerative lumbar spine disease and spondylodiscitis due to minimally invasive access to the spine, less blood loss compared to open procedures, decreased operative times, earlier mobilization, shorter hospital stays, and less postoperative pain [1-8]. In 2001, Pimenta introduced an innovative minimally invasive spine surgery that accessed the anterior lumbar spine, using a lateral, and transpsoas approach, which was published as a technical note in 2006 [9]. The method was initially described under the current name XLIF by Ozgur et al. in 2006 [10]. XLIF has proved itself to be an efficient means of treating various spinal pathologies, including degenerative spine disease (low-to-moderate central canal, lateral recess and/or foraminal stenosis, low-grade spondylolisthesis [Grade I-II], degenerative scoliosis, and degenerative disc disease), and spondylodiscitis [1,3,11,12]. The lateral approach allows for placement of a wide footprint intervertebral cage (18, 22, and 26 mm) with wide apertures to provide superior anterior column realignment as well as a healthy fusion environment without anterior and posterior longitudinal ligament (ALL and PLL) resection [13-15]. Indirect decompression by XLIF results from restoration of native disc height and subsequent stretching and tightening of the remaining annulus, causing elongation of the posterior longitudinal ligament, distraction of the ligamentum flavum, and ultimately leading to an increase of the epidural space [11,16]. In order to sustain indirect decompression, use of 26 mm cages was advised, as these cages compared to 18 mm and 22 mm wide cages significantly reduce cage subsidence in XLIF at mid-term follow-up [11]. Modification of this method, endoscope-assisted XLIF (EA-XLIF) has been described and considered particularly helpful for checking the lumbar plexus anatomy on the psoas surface, identifying the relationship between the peritoneum and the psoas muscle, positioning the shim into the disc space, removing the disc, and checking the quality of contralateral release and endplate preparation [17].

Unlike the traditional interbody fusions and approaches, the XLIF approach offers numerous advantages [18]. A general surgeon is not required for access, the need to retract or violate the peritoneum is eliminated, and the approach avoids mobilization of the great vessels, thereby avoiding the related risk of sexual dysfunction [19].

Cadaver studies defined the anatomy of the lumbar plexus and proposed an appropriate working space where dilators could be placed at each level of the lumbar spine [20,21]. When approaching the lumbar spine from L3, L2, or L1, the psoas muscle should be split into the ventral three-quarters of the vertebral body (VB) to avoid nerve injury [22]. There is risk to the genitofemoral nerve, if the psoas major muscle is split at L3 or L4 [23]. Placing the dilator or retractor in a posterior position may result in nerve injury, especially at L4-5 [23].

Surgical therapy of intervertebral disc degeneration is still a mainstay of treatment when conservative approach fails. Regenerative strategies for intervertebral disc disease such as tissue engineering with three-dimensional biomimetic scaffolds show great promise, although still in the experimental phase [24]. Several studies have reported good clinical and radiological outcomes for the XLIF procedure in the degenerative spine disease [25-27]. XLIF reduces the risk of nerve root lesions, postoperative radiculitis, and durotomies compared to posterior fusion techniques in revision surgeries [5]. In the large prospective, multicenter study by Philipps et al., significant improvements in visual analogue scale (VAS) and Oswestry disability index (ODI) scores for leg and back pain were observed in 107 patients, with successful correction of the Cobb angle from 20.9 to 15.2° [28]. Improved functional VAS and ODI outcomes and restored coronal deformity have been shown in systematic review with promising perspective for the treatment of regional and global degenerative spinal canal stenosis and scoliosis [25].

Beside its use in degenerative spine surgery, XLIF has found its application in operative treatment of spondylodiscitis in patients with epidural abscesses and neurological deficits who require surgery [1,29,30]. The excellent exposure in XLIF approach allows satisfactory debridement of the end-plate as well as fusion within the same approach and thus avoiding trans-thoracic or trans-abdominal approach [1]. Posterior approach is the most common approach for the treatment of spondylodiscitis in the lumbar spine, however despite allowing decompression of neural structures it carries a higher risk of neurological deficit and limit the exposure of disc/vertebral body which can result in inadequate fusion and a failure to correct a lordosis secondary to poor exposure and visualization [1] and destabilizes the spine even more as it requires a laminectomy in a spine that already has a disrupted anterior and middle column [29].

Most surgeons insert the interbody cage laterally and then insert pedicle or cortical screw and rod instrumentation posteriorly [31]. However, standalone cages have also been used to avoid posterior instrumentation [31]. Although recent study suggested that supplemental fixation did not significantly influence cage subsidence or segmental lordotic angle in patients who underwent XLIF [32], results of systemic reviews and meta-analysis suggest that addition of posterior instrumentation to transpsoas fusion is associated with decreased re-operations and cage movements [31]. Lateral interbody fusion (LIF) with percutaneous screw fixation can treat adult spinal deformity (ASD) in the coronal plane, but sagittal correction is limited [33]. Open posterior surgery with XLIF was associated with faster recovery, fewer complications, and greater relief of pain and disability compared to open posterior surgery alone [33].

Limitations of XLIF include neurovascular complications [4], anatomical limitations, subsidence, and loss of correction, declining the potential to restore spinal biomechanics sustainably [34]. Major factors prompted the development of minimally invasive (MIS) extreme lateral interbody fusion (XLIF; NuVasive Inc., San Diego, CA, USE) for the thoracic and lumbar spine, which include interbody stabilization and indirect neural decompression while avoiding major visceral/vessel injury as seen with anterior lumbar interbody fusion (ALIF), and to avert trauma to paraspinal muscles/facet joints found with transforaminal lumbar interbody fusion (TLIF), posterior lumbar interbody fusion (PLIF), and posterior-lateral fusion techniques (PLF) [4]. Although XLIF is associated with an increased prevalence of anterior thigh/groin pain as well as motor and sensory deficits immediately after surgery, pain and neurologic deficits decrease over time [35]. Although the majority of complications were minor, one survey reported a high complication rate of 18% with re-operation rate of 2.2% in Japan [36]. Recent multicentric retrospective cohort study has shown that the major complications rate was 0.7722% [37]. Relative contraindication to XLIF is bony lateral recess stenosis, which has shown to be an independent predictor for failure to achieve adequate spinal decompression through XLIF and thus may benefit from undergoing direct decompression [38].

Our objective is to determine the safety and efficacy of extreme lateral lumbar interbody fusion (XLIF) with supplemented instrumentation in degenerative spinal canal stenosis and spondylodiscitis. To the best of our knowledge, this is the first study that addresses clinical and radiological outcome of XLIF in patients with degenerative spinal canal stenosis and spondylodiscitis at a single institution.

## MATERIALS AND METHODS

Seven-two patients treated with XLIF between 2012 and 2018 were analyzed retrospectively. Data were gathered through review of patient’s electronic records and relevant imaging. Indications for XLIF included degenerative spine disease (including spinal canal stenosis with segmental scoliosis as well as uni- or bilateral neuroforaminal stenosis, adjacent segment disease with segmental scoliosis following spinal fusion, and instability of the spine following decompressive surgery) and spondylodiscitis in patients who underwent dorsal stabilization. In all cases, gadolinium-contrast enhancing magnet resonance imagining (MRI) of the spine as well as computed tomography (CT) of the spine was obtained. All patients received CT and X-ray of the instrumented region on the 1st day following surgery. Independent neuroradiologists verified neuroimaging.

Standard left lateral transpsoas approach with use of neuromonitoring was performed (NuVasive, San Diego, CA, USA^®^) [10,39]. All patients were fitted with a 10 degree lordotic intervertebral polyetheretherketone (PEEK) cage (Nuvasive^®^). The cages were 50, 55, or 60 mm in length, 18 mm in width and 8, 10, or 12 mm in height. All cages were filled with hydroxyapatite nanoparticles gel Nanogel^®^ (Teknimed, L’Union, France).

**FIGURE 1:**
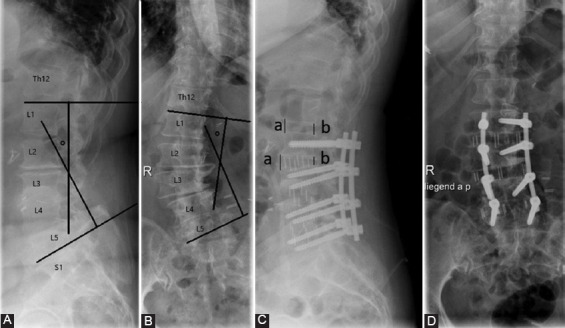
A. Preoperative lateral X-ray of the lumbar spine. Technique of assessment of L1-S1 angle (lumbar lordosis; angle between the upper endplate of L1 and upper endplate of S1 vertebra). B. Preoperative coronal X-ray of the lumbar spine. Technique of assessment of L1-L5 angle in the coronal plane (angle between the upper endplate of L1 and lower endplate of L5 in coronal plane). C. Postoperative lateral X-ray following three-level XLIF in L2/3, L3/4, L4/5 with supplemental fixation L2-5. Depiction of disc height assessment as an average of anterior and posterior disc height. D. Postoperative coronal X-ray shows increased disc height.

Follow-up comprised pain assessment with VAS and clinical examination. X-ray scans were obtained at 3, 12-, and 24-months following surgery, dynamic flexion-extension X-rays 6 months following surgery while CT scans were obtained in the period of 6-24 months. Radiographic analysis comprised measurement of fusion, L1-S1 sagittal lordotic angle (LL-lumbar lordosis), L1-L5 coronal angle, L5-S1 angle [40], and disc height. Disc height was measured an average of anterior and posterior disc heights [41] (Figure 1). Pelvic incidence (PI) was measured as the angle between the line joining the midpoint of the coxofemoral joint axis and the center of the S1 endplate and the line orthogonal to the S1 endplate [42]. For measurement of PI-LL value, PI was subtracted from the value of L1-S1 angle (LL-lumbar lordosis). Fusion was defined as the presence of trabeculae bridging bone formation at the anterior and/or posterior cortex of the involved vertebral bodies on the CT scan, and an interface between the cage and the vertebral endplate. Absence of such bridges was classified as non-fusion. The analyses were performed using SPSS statistical software, version 20 (SPSS Inc. IBM, USA). The value of *p* < 0.05 was considered to be statistically significant.

### Ethical statement

The local ethics committee at the University Hospital Marburg considered an ethical approval unnecessary for this pseudonymized retrospective analysis (Number of the ethical approval/Az: ek_mr_20_10_2020_2_pojskic).

### Statistical analysis

The analyses were performed using SPSS statistical software, version 20 (SPSS Inc. IBM, USA). The value of *p* < 0.05 was considered to be statistically significant. For variables such as gender, angle and disc height, mean was calculated with standard deviation (SD), for non-parametric variables (descriptive statistics of groups of degenerative spine disease/spinal canals stenosis and spondylodiscitis, influence of age, presence or absence of fusion as well as comparison between the groups) descriptive statistics was used for calculating frequencies in the two groups, using graphic diagrams as well as cross product and Pearson’s Chi Square test and Fisher Exact for testing of significance of differences of frequencies in the two groups. T-test was used for measurement of statistically significant difference between the means. For calculating differences between standard deviations, Leven’s Test for equality of variances was performed before t-test. If there was a statistically significant difference between the SDs, t test was not performed. Independent sample t test was used for comparison of different mean values between the two groups (spinal canal stenosis and spondylodiscitis) and paired samples t test for comparison of variables of two dependent samples for same patients in the different setting (e.g., comparison of parameters before and after surgery) which was used for determination of statistical significance.

## RESULTS

Seventy-two patients were included in the study. Patients’ characteristics and surgical management are summarized in Table 1. Forty male (55.6%) and 32 female (44.4%) patients were included in the study and medium age was 66.6 years. Forty-five patients (62.5%) were operated due to degenerative spine disease (spinal canal stenosis) and 27 patients (37.5%) with spondylodiscitis. The mean follow-up was 23 months. Three patients (4.2%, 2 with spondylodiscitis and 1 with spinal canal stenosis) died after more than 12 months following surgery. In 37 patients (51.4%) there were no previous surgeries on the lumbar spine, 35 or 48.6% underwent previous surgery via dorsal approach in the segment, which underwent XLIF.

**TABLE 1:**
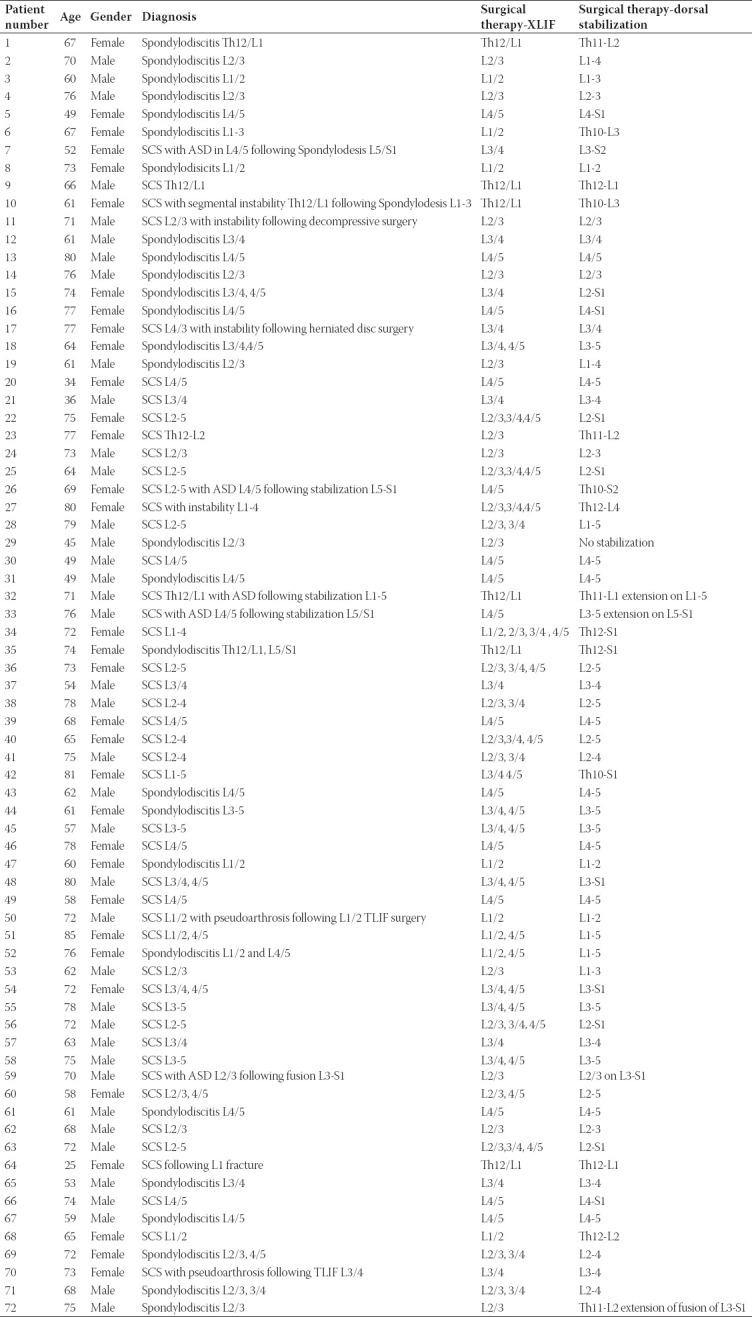
Patients’ characteristics and surgical management

### Symptoms and neurological deficits

All patients presented with back pain and radiculopathy. Spinal claudication was present in 41 patients (56.9%). Pain reduction was significant with preoperative VAS of 8.8 and postoperative VAS of 2.8 (paired samples t-test, t = 33.822, *p* < 0.05).

Forty-four patients (61.1%) had neurological deficits before surgery and only 12 patients (16.1%) had deficits following surgery. Twenty-one patients (29.2%) were neurologically unchanged, 6 patients (8.3%) worsened, and 45 patients (62.5%) improved. New postoperative thigh weakness was detected in 8 patients (11.1%), in 4 patients the symptoms completely resolved. From 6 patients who worsened, 4 had permanent postoperative left thigh weakness and two patients developed paraparesis to follow-up, both due to adjacent segment disease following dorsal spinal constructs of 5 segments with worsening of the spinal canal narrowing and compression of the cauda equina.

### Complications

Only one patient experienced a perioperative complication related to the lateral transpsoas approach with a retroperitoneal hematoma which was treated conservatively. Seventy-one patients received additional dorsal stabilization Complications related to the dorsal approach occurred in 10 patients (13.9%), in 3 patients (4.2%) hardware failure with screw malposition or screw breakage occurred, and in 7 patients wound healing problems (9.7%). In 17 patients (23.4%) adjacent segment disease occurred with the need of extension of the dorsal spinal construct during the mean period of 24.5 months (9-47.5 months). Mean surgery time was 142 min. Mean hospital stay was 14.3 days.

### Radiological outcome

Mean preoperative coronal L1-L5 angle was 4.28° compared to postoperative angle of 4.98° (t = –3.027; corr = 0.749, *p* < 0.05). Forty-four patients or 61.1% were hypolordotic before surgery (L1-S1<40°). Mean sagittal L1-S1 angle before the surgery was 36.2° and it increased to mean postoperative value of 38.09°. This lordosis correction was statistically significant (t = –3.292; corr = 0.852, *p* < 0.05). Lordosis correction was furthermore shown in the increase of L5-S1 angle (4.49° preoperative vs. 5.64° postoperative; t = –6.366; corr = 0.921; *p* < 0.05). Mean disc height increased significantly from 6.1 mm preoperative vs. 8.4 mm postoperative (t = –16.29; corr = 0.337; *p* < 0.05). Fusion in the XLIF segment occurred in 64 patients (89%). Only one patient developed symptomatic non-fusion (pseudoarthrosis). Mean preoperative pelvic incidence (PI) was 57.1° (SD ± 1.8) compared to postoperative PI of 56.4° (SD ± 27.2). Mean preoperative PI-LL (pelvic incidence minus lumbar lordosis L1S1) value was 21.15° (SD ± 3.45) and mean postoperative PI-LL value was 18.35° (SD ± 14.4). Reduction of PI-LL value was statistically significant (*p* < 0.05). In 19 patients postoperative PI-LL value of <10° was achieved; however, their clinical outcome was not favorable compared to other 52 patients.

One level XLIF was performed in 49 patients (68%), two level in 15 patients (20.8%), three level in 7 patients (9.7%), and four level in 1 patient (1.4%). All XLIF-levels are summarized in Table 2. Most common level was L4/5 (in 32 patients or 44.4%) followed by L3/4 (in 29 patients or 40.3%), L2/3 (in 26 patients or 36.1%), L1/2 (in 10 patients or 13.9%), and Th12/L1 (6 patients or 8.3%). Neurological and radiological outcome did not differ between patients with single level and multiple level XLIF. Dorsal stabilization was performed in 71 patients: One segment in 25 patients, two segments in 12 patients, three segments in 7 patients, and four and more segments in 27 patients.

**TABLE 2:**
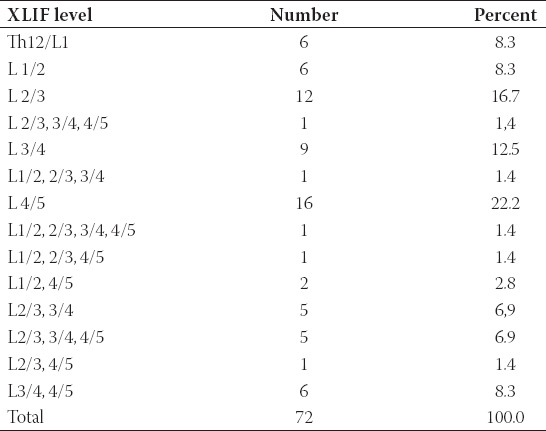
XLIF-levels

### Comparison between patients with spinal canal stenosis and spondylodiscitis

Patients with spinal canal stenosis had longer follow-up of 29.2 months compared to 12.8 months in patients with spondylodiscitis with statistical significance (t = –3.005; *p* < 0.05). Baseline characteristics (age, gender, and preoperative neurological deficits) did not differ between the two groups.

Radiological outcome did not differ between the two groups of patients with degenerative spinal disease (spinal canal stenosis) and spondylodiscitis. Mean values of preoperative and postoperative parameters (PI, LL, PI-LL, value, L1-S1 and L5-S1 angle) were not statistically significantly different between the two groups. The mean values of the important radiological parameters are summarized in Table 3.

**TABLE 3:**
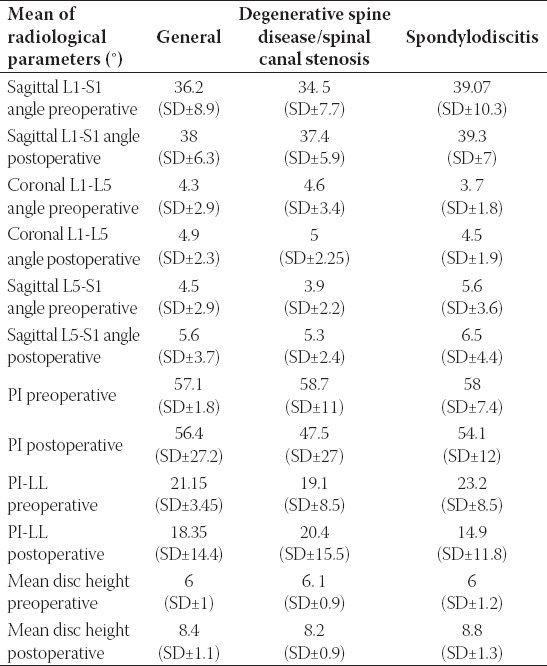
The mean values of the important radiological parameters

Patients with spondylodiscitis had statistically significant higher rate of worsening of neurological deficits following surgery (5 patients vs. 1 patient with spinal canal stenosis, Chi-Square = 5.867, *p* < 0.05). Patients with spondyodiscitis had more frequent previous surgery on the spine (22 vs. 15. patients, Pearson’s Chi-square = 15.660, *p* < 0.05). Non-fusion occurred more frequently in patients with spondyodiscitis (6 vs 2. patients with spinal canal stenosis, independent samples test/Levene’s test for equality of variances and t-test for equality of the means, t = 6.846, *p* < 0.05). Patients with spondylodiscitis had a longer hospital stay (19.5 vs. 11.1 days, t = 3.987; *p* < 0.05). Adjacent segment disease developed more frequently in patients with spinal canal stenosis (16 vs. 1. patients, Chi-square: 11.954, *p* < 0.05). Surgery times were longer in patients who underwent surgery for spinal canal stenosis compared to patients with spondylodiscitis (158.8 vs. 117.8 min, t = –2.481, *p* < 0.05).

## DISCUSSION

XLIF was described as being an effective minimally invasive method for degenerative spinal canal stenosis [43,44] as well as for spondylodiscitis [1,29]. Beside the classical indications for XLIF as degenerative lumbar spine diseases with scoliosis and uni- and bilateral neuroforaminal stenosis, its use in spondylodiscitis with [29] or without [1] supplemental fixation was recently described. One of the advantages of XLIF in treatment of infectious spine diseases is that it affords adequate exposure to the vertebral bodies and discs to aggressively debride necrotic and infected tissue [29]. Although clinical application of standalone XLIF is well known [1,45], XLIF is often being used in addition to dorsal stabilization in order to prevent cage sinking and improve fusion [1], where it is associated with faster recovery, fewer complications, and greater relief of pain and disability compared to dorsal surgery alone in treatment of adult spinal deformity [33]. To the best of our knowledge, this is the first study that evaluates the clinical and radiological outcome of patients treated with XLIF due to degenerative as well as infectious disease of the lumbar spine treated in single neurosurgical unit.

Pain reduction was significant as shown in the previous studies [2,25,29,43]. Recent literature review weighted average mean of preoperative VAS pain scores of 6.8, compared to a postoperative VAS score of 2.9 (*p* < 0.0001) [25]. Neurological outcome in 91.7% of patients who were unchanged or improved following surgery was good compared to reported data [2,6,12,44]. Thirty-five patients (48.6%) underwent previous surgery in the segment that underwent XLIF. XLIF has been shown to be an effective fusion technique in revision surgery that allows valid arthrodesis by avoiding scarred tissue due to earlier surgical approaches. It reduces the risk of nerve root lesions, postoperative radiculitis, and durotomies compared to posterior fusion techniques [5].

Decrease of VAS and ODI (Oswestry Disability Index) in terms of favorable clinical outcome has been shown in numerous studies by Khajavi et al. [46] (160 patients), Formica et al. [6] (39 patients), Tohmet et al. [47] (140 patients), Philipps et al. [28] (107 patients), Rodgers et al. [15,48] (600 patients in the first study and 63 in the latter one), Malham et al. [13] (30 patients), Paterakis et al. [7] (12 patients), Timothy et al. [1] (14 patients), Attenello et al. [49] (22 patients), Tamburelli et al. [27] (21 patients), Campbell et al. [12] (18 patients), Tessitore et al. [50] (20 patients), Blizzard et al. [29] (11 patients), Lykissas et al. [35] (451 patient), and Isaacs et al. [51] (29 patients with XLIF). In lumbar interbody fusion, MIS-TLIF (minimally invasive transforaminal interbody fusion) had better ODI, VAS pain, and complication rate when compared to XLIF with direct and indirect meta-analysis methods; however, in terms of fusion rates, there were no differences between the two techniques [44]. Radiological outcome showed similar results in patients with degenerative spinal canal stenosis and spondylodiscitis. Improvement of regional lordosis (increase of sagittal L1-S1 angle of 36.2° preoperative vs. 38.09° postoperative) showed consistence with the previous studies [7,25,29,30]. However, measurement of the regional and segmental coronal Cobb angles did not show correction but rather a slight angle increase (4.28° preoperative vs. 4.98° postoperative) which implicates an insufficient correction of lumbar scoliosis [43]. In studies which interrogated use of XLIF in patients with coronal deformity as the main indication, XLIF was shown to be an efficacious procedure for achieving the coronal alignment [52]. However, these improvements were lower in the following studies probably due to the fact that the patient selection was not limited to only those with scoliosis [2]. Due to additional dorsal stabilization and release of the posterior elements with neuroforaminal decompression, as well as due to high fusion rates, this lack of coronal correction did not have clinical implications. Patients with satisfactory fusion rates and sustained restoration of lordosis and disc height have shown to have positive clinical outcomes [6,45]. The weighted average preoperative and postoperative coronal segmental Cobb angles in the literature were 3.6 and 1.1° and weighted average preoperative and postoperative coronal regional Cobb angles were 19.1 and 10.0°, respectively [25]. Disc height increased following cage implantation from 6.1 mm preoperative to 8.4 mm postoperative. Disc height increase leads to indirect decompression of the nerve in the foramina and leads to a restoration of segmental lordosis and scoliosis and is consistently reported to occur following XLIF in degenerative and infectious spine disease [2,45,53,54]. Fusion rates show heterogeneity, which is based on the technique of fusion assessment and influenced by varying lengths of the follow-up (89-100%) [2,5,6,55]. A recent study reported a 2-year-fusion rate of 85.71% without differences between standalone construct compared to supplemental fixation [32]. Factors thought to contribute to cage subsidence are the narrower 18 μm cages, osteoporosis, the use of bone morphogenetic protein (BMP-2), the use of standalone cages, and iatrogenic endplate violation [56]. Taller cage height, narrower cage width, and shorter cage length were significantly associated with increased risk of cage settling more than 4 mm at 12 months postoperatively [47]. In patients with no cage settling immediately postoperatively, risk of settling more than 4 mm at 12 months was 6.8 times greater with narrower cages [47]. As previously mentioned, addition of posterior instrumentation to transpsoas fusion is associated with decreased re-operations and cage movements [31]. Titanium cages were associated with lower subsidence rates than PEEK cages [57]. Usage of rh (recombinant human) BMP-2 was also robustly associated with higher endplate subsidence [57]. The formula of PI minus LL (PI - LL) offers an estimate of the lordosis required for a given PI value and tries to quantify the mismatch between pelvic morphology and the lumbar curve. Schwab et al. [58,59] suggested that a PI - LL < 10° represents satisfactory spinopelvic alignment and incorporated this into an adult spinal deformity classification [60]. An excessive PI-LL mismatch (PI-LL > 10°) is more likely to lead to the development of adjacent segment disease and the requirement of a revision surgery [61]. In our study, postoperative PI-LL value was 18.35 for the entire cohort (20.4 for degenerative spine disease and 14.9 for spondylodiscitis) and although the correction was statistically significant compared to preoperative value, it did not correlate with the patient clinical outcome. One of the reasons could be that PI-LL value has shown its application in adult spinal deformity surgery, which requires larger spinal constructs, which often include thoracic and lumbar spine and more invasive posterolateral surgery than the mono-or multisegmental XLIF surgery. Use of regional lumbar lordosis has been proposed as a more accurate assessment for quantification of normolordosis, since Furthermore, patients with adult degenerative spine disease and scoliosis are older than the patients with adult deformity [62]. Women are shown to have larger normal PI-LL [63]. Recent studies have shown that patients with a large PI sometimes have good surgical results, even with a postoperative PI-LL>10° [64] in up to 23% of patients following extensive surgery for degenerative scoliosis [65]. One recent study found that ideal PI-LL may be between 10° and 20° in ADS patients after long posterior instrumentation and fusion [62]. An optimum PI-LL has been shown inconsistent in that it depends on the individual PI [64]. Two studies examined the relationship between parameters of spinopelvic alignment and standalone XLIF surgery [54,66]. The studies have found that XLIF improved scoliosis and segmental lordosis and was associated with significant clinical improvement in patients with lumbar degenerative disc disease [66]. However, XLIF did not change overall lumbar lordosis or significantly alter pelvic indices associated with sagittal balance [66]. For the lower lumbar spine, it is difficult to obtain a lordosis more than 10 degrees with stand-alone XLIF for correcting adult spinal deformity [67]. Therefore, it is thought that correction such as osteotomy or compression technique to the posterior fusion may be necessary during the second stage surgery [67]. Other studies also showed no significant change in the overall coronal or sagittal plane alignment of the lumbar spine [14]. End-plate breach was common at the instrumented disc levels; however, it was nonprogressive in most of the cases, and did not affect the fusion or alignment at the instrumented levels [14]. Tessitore et al. have shown that mono- and bisegmental lordosizing fusion techniques, as XLIF and TLIF, are able to restore disc height and improve segmental lordosis [68]. However, they do not allow restoration of sagittal balance or improvement of compensatory mechanisms [68]. In our study, the mean surgery time was with 142 min. In the literature operative time reported to vary from 125.6 min [25] to 218 min [53]. Longer OR time could be explained with higher number of multilevel XLIF. Mean hospital stay was 14.3 days, which was longer than in the previous studies [1,55]. This could be explained with larger percentage of patients with spondylodiscitis who received i.v. antibiotic therapy and screening for further diseases as well as prolonged stay at the hospital due to postponed discharge for social reasons. Our study showed a low complication rate with only one patient experiencing postoperative hematoma, which was treated conservatively. There were no major complications. The overall complication rate has been shown to be high and range from 18% [36] to 23% [69]. Most frequent major complications are major vascular injury, bowel injury, and surgical site infection (0.03%, 0.03%, and 0.7%, respectively) with overall re-operation rate of 2.2% [36]. Vertebral body fracture and contralateral nerve injury were reported in 3.7% of patients [69]. Transient ipsilateral thigh numbness, pain, and/or hip flexor weakness are a frequent postoperative finding most commonly when the L4-L5 level is instrumented and it was described in the literature to range from 4.4% [2,36], 18% [6], 19.4% [69] to 25.7% [6,14,53]. This symptom is considered by some authors as minor complications [69] and by some authors as accepted approach related symptom [6].

Differences between outcome of patients with spinal canal stenosis and spondylodiscitis seem to be more related to the pathology and the dorsal approach than to the XLIF method itself. Patients with spondylodiscitis showed a higher rate of worsening of neurological deficits following surgery and a higher incidence of non-fusion. The most common postoperative deficit was ipsilateral thigh weakness and its higher incidence in patients with spondylodiscitis could be explained with infection affecting paravertebral muscles. Higher incidence of non-fusion could be explained with shorter follow-up as well as worsened bone substance due to infection.

Limitations of our study are its retrospective nature and relatively small number of patients; however, prospective studies with larger number of patients are needed for further evaluation of the application of XLIF in patients with spinal canal stenosis and spondylodiscitis.

## CONCLUSION

Extreme lateral interbody fusion (XLIF) with supplemented instrumentation is a safe method for surgical therapy of degenerative spine disease (spinal canal stenosis with segmental scoliosis and bilateral neuroforaminal stenosis) and spondylodiscitis. Patients with degenerative and infectious spine disease show similar radiological outcome following XLIF. Patients with spondylodiscitis show a higher rate of worsening of neurological deficits following surgery and a higher incidence of non-fusion so the indication to apply XLIF in these patients should be carefully evaluated.
